# Undifferentiated carcinoma with osteoclast-like giant cells of the pancreas: a rare case report and review of the literature

**DOI:** 10.3389/fsurg.2025.1584200

**Published:** 2025-07-18

**Authors:** Lu Si, Yihan Liu, Zhiyu Lin, Qiuwen Qin, Yuxu Zhang, Demao Deng, Zhao Lu

**Affiliations:** International Medical Department, People’s Hospital of Guangxi Zhuang Autonomous Region, Nanning, China

**Keywords:** undifferentiated carcinoma, osteoclast-like giant cells, pancreas, computed tomography, immunotherapy

## Abstract

**Background:**

Undifferentiated carcinoma with osteoclast-like giant cells of the pancreas (UC-OGCP) is an exceedingly rare malignant tumour that accounts for less than 1% of all pancreatic nonendocrine neoplasms.

**Case presentation:**

We present a case of UC-OGCP in a 46-year-old male patient who was referred to our hospital after the incidental identification of a cystic tumour in the pancreas. Computed tomography (CT) revealed a 1.4 × 1.4 × 1.6-cm mass in the pancreatic head. Surgical resection was performed because it was difficult to determine the degree of malignancy of the tumour through clinical assessment. Intraoperative frozen-section pathology confirmed that the tumour was a malignant neoplasm. Pancreaticoduodenectomy was subsequently conducted. Histopathological studies confirmed the diagnosis of UC-OGCP.

**Conclusion:**

The clinical symptoms of UC-OGCP are nonspecific, and there are no distinct serological markers. Diagnosis relies primarily on endoscopic biopsy or postoperative pathology. We report this case of UC-OGCP and provide a literature review on the clinicopathological features, differential diagnosis, treatment, and prognosis of UC-OGCP, aiming to improve the understanding of this disease.

## 1 Introduction

Undifferentiated carcinoma is a rare and highly aggressive subtype of pancreatic cancer, and undifferentiated carcinoma with osteoclast-like giant cells of the pancreas (UC-OGCP) accounts for less than 1% of all pancreatic cancer cases (1). UC-OGCP is composed of three main cell types, namely, nonneoplastic osteoclast-like multinucleated giant cells (OGCs), mononuclear histiocytes (MCHs), and neoplastic mononuclear cells, that can appear in different proportions, and the distribution of these cells affects prognosis; among these cell types, mononuclear tumour cells can include spindle-shaped cells that resemble epithelioid cells and pleomorphic giant cells, and the presence of these cell types is associated with more malignant tumours (2). The authors reasoned that UC-OGCP likely originates from epithelial and mesenchymal cells, and it is considered a rare morphological variant of pancreatic ductal adenocarcinoma (PDAC) (3). Although this condition was first described by Rosai (4) in 1968, only a limited number of cases have been reported, and the histological features and other characteristics of these tumours remain unclear or controversial. Imaging studies typically reveal the presence of a large mixed cystic or solid mass in the pancreas (5). The preoperative diagnosis of UC-OGCP presents significant challenges for clinicians, and diagnosis primarily depends on endoscopic biopsy or postoperative pathology (6). Here, we report a case of UC-OGCP and provide a literature review on the clinicopathological features, differential diagnosis, treatment, and prognosis of this disease, aiming to improve the understanding and awareness of this rare tumour.

## 2 Case presentation

A 46-year-old man was referred to our hospital after the incidental identification of a cystic tumour in his pancreas. The patient denied symptoms such as nausea, vomiting, diarrhoea, constipation, abdominal pain, appetite changes, or bloating. Moreover, his medical history was unremarkable, with no records of pancreatitis or solid organ malignancy. The patient also reported no allergies or significant social or family medical history, and physical examination revealed no obvious abnormalities. Serum biochemistry results revealed an elevated CA-19-9 level of 76.4 U/ml (normal range: <0–34 U/ml), while the patient's alpha-fetoprotein, carcinoembryonic antigen, carbohydrate antigen 125, carbohydrate antigen 15–3, and CEA levels were within normal ranges.

**Figure 1:**
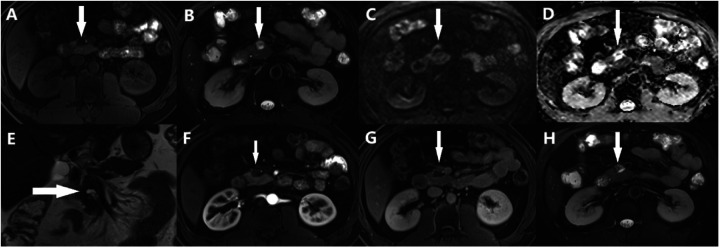
Abdominal MRI showed a solid lesion with cystic lesion, **(A)** T1WI showed hypointensity (arrows), **(B)** T2WI showed high-intensity (arrows), **(C)** DWI showed iso-intensity (arrows), **(D)** ADC showed high-intensity (arrows), **(E)** Coronal T2WI showed a ring-shaped low signal on T2WI at the periphery of the lesion (arrows), **(F-H)** the lesion had obvious progressive enhancement.

**Figure 2:**
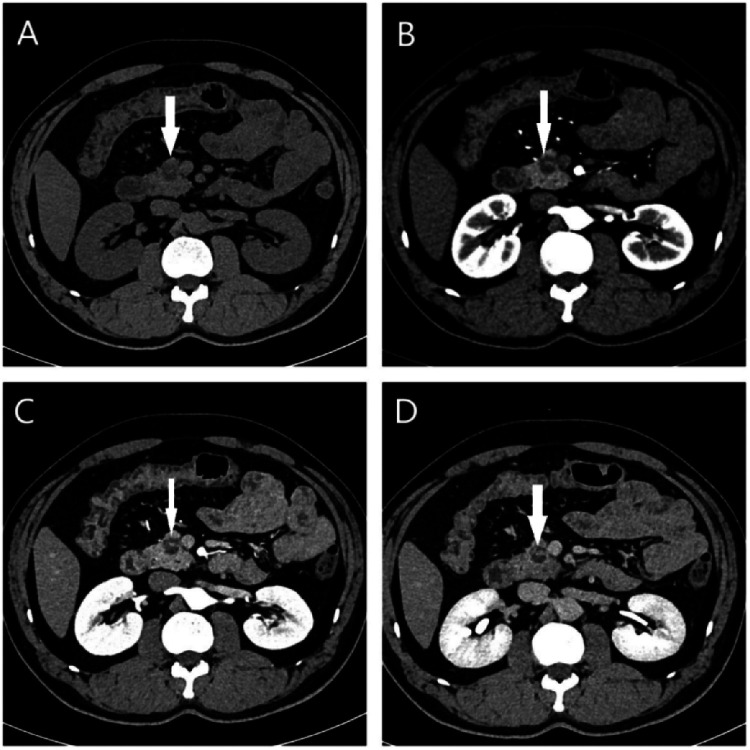
**(A)** On the plain CT scan, a small lesion with heterogeneous density and indistinct borders located in the pancreatic head (white arrow) were observed. Contrast-enhanced axial CT images showed the mass (white arrow) with heterogeneous enhancement in the **(B)** arterial, **(C)** venous and **(D)** delay phase.

**Figure 3:**
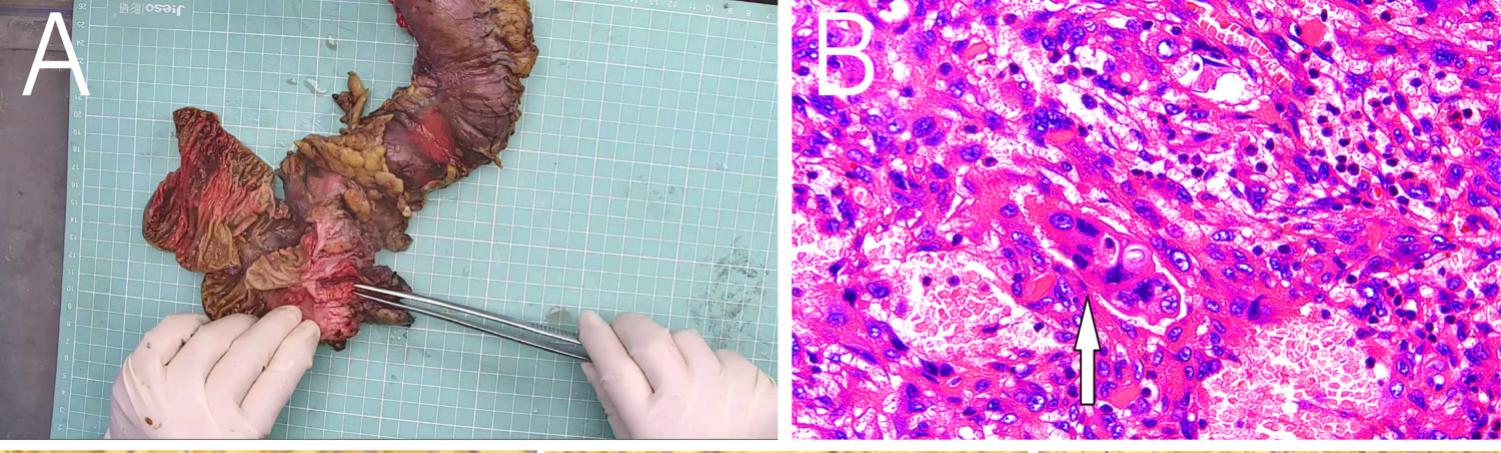
**(A)** Postoperative mass specimen of undifferentiated carcinoma with osteoclast-like giant cells of the pancreas, **(B)** The white arrow indicates osteoclast-like giant cells. The image is magnified 400 times.

**Figure 4:**
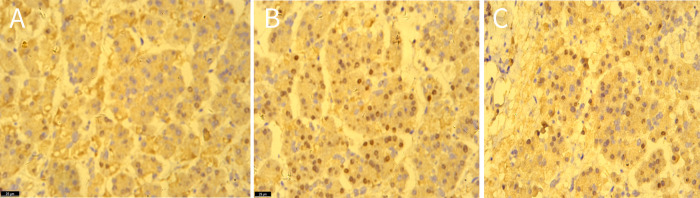
The immunohistochemical staining showed the expression of CD163 **(A)**, P53 **(B)**, ki-67 **(C)** in tissues, original amplification ×400.

MRI revealed a space-occupying lesion in the head of the pancreas; the shape was regular, the boundary was clear, and the internal intensity was uneven. T1-weighted imaging (T1WI) showed hypointensity, T2-weighted imaging (T2WI) showed high intensity, diffusion-weighted imaging (DWI) showed isointensity, and the ADC showed high intensity (Figures 1A–D). Coronal T2WI revealed a ring-shaped low signal intensity at the periphery of the lesion, which suggested the possibility of haemosiderin deposition (Figure 1E). The mass showed heterogeneous peripheral progressive enhancement on T1 fat-sat postcontrast imaging (Figures 1F–H). Further investigation via enhanced computed tomography (CT) confirmed that the lesion was approximately 1.4 × 1.4 × 1.9 cm^3^ in size, and an enhanced CT scan revealed that the solid part of the lesion in the arterial stage showed uneven enhancement and that the extent of enhancement of the solid part decreased in the delayed period (Figures 2A–D). The preoperative evaluation suggested that the lesion was a cystic neoplasm, but it was unclear whether the lesion was benign or malignant. A discussion was had with the patient about the choice of surveillance or surgical resection, and the patient preferred the latter option. Thus, the patient underwent an elective exploratory laparotomy. The lesion was determined to be a malignant tumour intraoperatively via the use of frozen sections. The patient underwent standard pancreaticoduodenectomy. Histological examination revealed that the tumour comprised nonneoplastic osteoclast-like giant cells and neoplastic pleomorphic cells (Figure 3). Additionally, immunohistochemical staining revealed that the tumour was positive for CD163, P53, Ki-67 (Figure 4), CD68, INI-1, SATB2, PMS2, and MSH6 expression and negative for CK, CK7, CEA [M], and CAM5.2 expression.

Appropriate imaging studies did not reveal metastases. The final pathological stage of the tumour was the Union for International Cancer Control pT1cN0M0 stage I AJCC 8th edition. Following surgery, the patient's progression was uneventful, and imaging data revealed no evidence of recurrence at the 6-month follow-up visit.

## 3 Discussion

UC-OGCP is an extremely rare and highly aggressive type of pancreatic malignancy (7). The 2010 WHO classification of pancreatic tumours considers UC-OGCP to be a subtype of PDAC, although its pathogenesis remains largely unclear (8). UC-OGCP can affect patients across a wide range of ages, with no apparent sex predilection (9). Owing to the lack of distinctive clinical symptoms, physical signs, or serological markers, patients are often diagnosed at an advanced stage of disease, which contributes to the generally poor prognosis (10). According to previous reports, patients commonly present with nonspecific gastrointestinal symptoms, including abdominal pain, distension, fatigue, jaundice, and weight loss (11). Tumour marker levels are typically within normal ranges, although elevated CA199 levels are observed in some patients (12). In the present case, the patient's pancreatic lesion was incidentally discovered through imaging, which enabled timely intervention. As a result, the tumour was identified at an early stage, and the patient experienced a favourable prognosis.

The imaging features of UC-OGCP provide valuable insights into tumour characteristics. On CT, UC-OGCP lesions generally appear as well-defined cystic or cystic‒solid masses, often exceeding 8 cm in diameter, and they are typically located in the body or tail of the pancreas (13). UC-OGCP is frequently associated with internal necrosis and haemorrhage, with occasional calcifications (14). When located in the pancreatic head or neck, UC-OGCP often causes dilation of the pancreatic and bile ducts (15). After contrast enhancement, visible enhancement is observed in the solid areas, septations, and lesion margins (16). On MRI, UC-OGCP tumours commonly show mixed signal intensities. T1WI reveals areas of low to slightly low signal intensity that are occasionally interspersed with patchy regions with high signal intensity. T2WI displays a heterogeneous mix of high and low signal intensity, with visible septations and cystic cavities of irregular thickness; fluid‒fluid levels are frequently observed within these cavities, and a rim of low signal intensity is often observed around the lesion margins (17). In most cases, the tumour exhibits low signal intensity on DWI, likely due to haemosiderin deposits from the phagocytosis of erythrocytes by osteoclast-like giant cells (18). On PET-CT, UC-OGCP lesions typically show high uptake of fluorodeoxyglucose (FDG), reflecting high metabolic activity that is consistent with the rapid growth of tumour cells (19).

The definitive diagnosis of UC-OGCP relies primarily on endoscopic biopsy or postoperative pathological examination of three main types of cells: nonneoplastic OGCs, neoplastic mononuclear cells, and MCHs (20). Mononuclear tumour cells can include spindle-shaped cells that resemble epithelioid and pleomorphic giant cells (21). OGCs contain eosinophilic cytoplasm and multiple nuclei and are generally located in haemorrhagic and necrotic regions (22). Immunohistochemical staining has revealed that MCHs express CD163, which is a marker of tumour-associated macrophages, whereas OGCs and some MCHs express CD68 (23). In UC-OGCP, elevated Ki-67 expression is predominantly observed in the mononuclear tumour cell population, particularly in histiocyte-like sarcomatoid cells (HSCs) and pleomorphic giant carcinoma cells (PCs). These cell types exhibit marked proliferative activity, with Ki-67 labelling indices typically ranging from 15% to 45%, which indicates the high degree of malignancy and aggressive biological behaviour of these cells (11). In contrast, OGCs, although present within the tumour microenvironment, display low Ki-67 expression. These cells are generally considered to be nonneoplastic, macrophage-derived reactive cells that lack proliferative potential (24). Thus, the aggressive nature of UC-OGCP is driven primarily by mononuclear tumour components with high Ki-67 expression, especially HSCs and PCs. The elevated proliferative index of these cells is strongly correlated with rapid tumour progression, a propensity for metastasis, and poor clinical outcomes.

Currently, there is no standard protocol for treating UC-OGCP. For early-stage cases, surgical resection is the primary treatment option (25). However, owing to the lack of specific clinical features, most patients are diagnosed at an advanced stage of disease or after metastasis has occurred. UC-OGCP, which is a variant of PDAC, has a striking genetic similarity to PDAC (26). Recent genomic studies suggest that UC-OGCP shares key molecular alterations with PDAC, including recurrent mutations in KRAS, TP53, and SMAD4 (27). Given the genomic similarities of UCOGCP and PDAC, the use of therapeutic strategies that are effective in treating PDAC for the treatment of aggressive UC-OGCP, particularly in the treatment of chemotherapy-resistant tumours, should be explored. In certain locally advanced, unresectable cases, gemcitabine may be recommended as palliative therapy, although the available data are limited, and no clear consensus has been established (28). Studies have demonstrated that PD-L1 is expressed in tumour cells in approximately 63% of UC-OGCP patients, and PD-L1 expression is strongly associated with a poor prognosis, potentially offering valuable insights for the development and optimization of treatment strategies (29). PD-L1 expression has been associated with poor prognosis in various cancers, including PDAC, where its presence is associated with immune evasion and resistance to therapy (30). Several studies have highlighted the prognostic value of PD-L1 expression and demonstrated that high PD-L1 levels are correlated with advanced disease stages and worse overall survival in multiple cancer patient population. Notably, Hrudka et al. (31) reported that patients with PD-L1-negative UC-OGCP exhibit a more favourable prognosis than patients with PD-L1-positive UC-OGCP. Obayashi et al. (32) reported a durable response to pembrolizumab in a patient with metastatic UC-OGCP, emphasizing the potential of immune checkpoint blockade in patients with high PD-L1 expression. Similarly, Besaw et al. (33) reported a favourable response to anti-PD-L1 therapy in a patient with recurrent UC-OGCP, further supporting the clinical importance of immune modulation in managing this aggressive variant of pancreatic cancer.

Our study highlights the importance of further investigation when cystic‒solid pancreatic lesions are identified, especially in patients with elevated tumour marker levels. Optimal diagnostic procedures include EUS-guided fine-needle aspiration or fine-needle biopsy, which allows the use of haematoxylin and eosin staining and immunohistochemical analysis to inform subsequent treatment decisions. This approach can facilitate the early diagnosis and effective management of malignancies. In our patient, the tumour was discovered at an early stage during routine examination, allowing prompt intervention, which contributed to a favourable prognosis.

## 4 Conclusion

UC-OGCP is a rare malignant tumour that typically presents with nonspecific clinical symptoms. While imaging techniques such as CT and MRI can assist in the diagnostic process, definitive diagnosis relies on pathological examination and immunohistochemical analysis. Surgical intervention remains the mainstay of treatment. For patients who are ineligible for radical resection or those with metastatic disease or postoperative recurrence, adjuvant therapies, including radiotherapy and chemotherapy, may be employed. This study presents a confirmed case of UC-OGCP, reviews its clinical data and integrates insights from the literature to discuss the clinicopathological features, key points in diagnosis and differential diagnosis, treatment strategies, and prognosis of UC-OGCP. The aim of this study was to advance the understanding and awareness of this rare condition.

## Data Availability

The original contributions presented in the study are included in the article/Supplementary Material, further inquiries can be directed to the corresponding authors.
